# Long term maintenance of glucose and lipid concentrations after Roux-en-Y gastric bypass

**DOI:** 10.20945/2359-3997000000047

**Published:** 2018-05-07

**Authors:** Fernanda Cristina Carvalho Mattos Magno, Priscila Alves Medeiros de Sousa, Marcelo Paiva Rodrigues, Lícia Lopes Pio Pereira, José Egídio Paulo de Oliveira, Eliane Lopes Rosado, João Régis Ivar Carneiro

**Affiliations:** 1 Universidade Federal do Rio de Janeiro Universidade Federal do Rio de Janeiro Rio de Janeiro RJ Brasil Universidade Federal do Rio de Janeiro (UFRJ), Rio de Janeiro, RJ, Brasil; 2 Instituto Fernando Luiz Barroso Rio de Janeiro RJ Brasil Instituto Fernando Luiz Barroso, Rio de Janeiro, RJ, Brasil

**Keywords:** Bariatric surgery, weight regain, lipemia, glycemia

## Abstract

**Objective:**

Roux-en-Y gastric bypass (RYGB) reduces body weight and the comorbidities associated with obesity. The aim of this study was to evaluate whether glucose and lipid profiles were maintained during a 5-year follow-up period after RYGB.

**Subjects and methods:**

Anthropometric and laboratory data from 323 patients who had undergone this operation were analyzed. Differences in laboratory variables between the baseline and 12, 24, 36, 48 and 60 months postoperatively (PO) were assessed using a one-way ANOVA test to compare the three groups. Delta significance using one-way ANOVA was performed to assess anthropometric variable in the postoperative period (p < 0.05).

**Results:**

77 patients (24%) were included in Group 1 (G1), 101 (32%) in Group 2 (G2), and 141 (44%) in Group 3 (G3). The majority of patients, 71.7% in G1, 82.8% in G2, and 70% in G3, showed high triglycerides (TG) before surgery. A decrease in weight loss was observed in all groups followed by an increase in body weight in G2 and G3 at 36, 48 and 60 months. Laboratory results for G1, G2 and G3 showed no significant differences between groups at baseline and during the post-operative period.

**Conclusion:**

Our results suggest that weight regain after RYGB has no significant impact on the long-term evolution of the lipid profile and glycemia.

## INTRODUCTION

Obesity is considered a risk factor for the development of various comorbidities, including impaired glucose homeostasis and cardiovascular disease (1). Roux-en-Y gastric bypass (RYGB) is the most commonly performed surgical treatment for obesity; however, although it is well documented that RYGB results in weight loss and metabolic improvements, some degree of weight regain has been observed 2–3 years after surgery (2), supporting the hypothesis that obesity is a chronic, incurable and progressive disease (3).

RYGB includes the anatomical diversion of the upper gastrointestinal tract. These changes in intestinal anatomy are thought to alter the function of the nutrient-driven enteroendocrine system, causing beneficial effects on glucose homeostasis that are independent of weight loss itself (4). In addition, weight loss elicits metabolic and cardiovascular benefits after RYGB, but evidence suggests that surgery might also activate weight-independent lipid amelioration mechanisms (1). Garcia-Marirrodriga and cols. (5) reported significant associations between lipid subfractions and excess weight loss after RYGB. However, a recent study conducted by Bradley and cols. (6) does not support the notion of clinically meaningful, weight loss-independent effects of RYGB surgery on β cell function and insulin sensitivity in non diabetic obese subjects after they have experienced considerable weight loss.

Weight loss results in improvements in glycemia and lipemia, and this is most evident in patients who have undergone RYGB (7). However, an elegant review by Shah and cols. (8) showed that the initial improvements seen following surgery decreased over a long-term follow-up period, possibly because of weight regain or unsatisfactory weight loss. Since weight gain can be considered a risk factor for dyslipidemia and changes in glucose levels, the aim of this study was to evaluate whether glucose and lipid profiles were maintained during a 5-year follow-up period after RYGB.

## SUBJECTS AND METHODS

### Subjects

This retrospective study was conducted by examining the medical records of 323 patients who underwent RYGB in a private clinic in Rio de Janeiro from 2000 to 2007 and maintained regular postoperative follow-up visits for at least 24 months after surgery. Personal, anthropometric and laboratory data were obtained from the medical records. This study was approved by the Research and Ethics Committee of the Clementino Fraga Filho University Hospital (176/09).

Three groups were classified according to the rate of inclination of each individual weight over time using linear regression slope for subjects with at least 36 months follow-up. The slope was obtained from the first 24 months after surgery, when patients’ body weight is expected to stabilize. Negative slopes were considered G1 (patients without weight regain). Positive slopes (variation between 0 and 2 kg) were considered G2 (patients who might or might not regain weight). Positive slopes (over to 2 kg) were considered G3 (patients with weight regain).

### Anthropometric and laboratory assessment

Anthropometric variables which were assessed included height and weight measured during the preoperative period and at 12, 24, 36, 48 and 60 months PO. Body mass index (BMI) was calculated by dividing the body weight in kilograms (kg) by the square of the height in meters (m^2^) (9). Total body mass and stature were measured following Gibson, using a mechanical anthropometric balance (Welmy^®^) with a maximum capacity of 300 kg, divided by 100 g, and the stadiometer from the anthropometric balance with a scale of 0.1 cm (10). The patients were barefoot and wore minimal clothing when weighed.

Laboratory parameters assessed included total cholesterol (TC), low-density lipoprotein cholesterol (LDLc), high-density lipoprotein cholesterol (HDLc), triglycerides (TG) and glucose. These were measured during the preoperative period and at 12, 24, 36, 48 and 60 months PO. TC, HDLc, TG and glucose were determined by the enzymatic-colorimetric method (11). Concentrations of LDLc were calculated based on Friedwald's equation (12). Concentrations of serum lipids that were considered normal were those provided in the guidelines of the National Cholesterol Education Program – Adult Treatment Panel III (NCEP/ATPIII): CT < 200 mg/dL, TG < 150 mg/dL, HDLc > 40 mg/dL e LDL-c < 130 mg/dL (13). A concentration of < 100 mg/dL was considered normal for fasting blood glucose (14).

### Statistical analysis

Means and standard deviations (SD) were determined for quantitative data. All data were assessed for normality using the Kolmogorov-Smirnov test. The Chi-square test was used for the comparison of proportions of abnormal values in TC, LDLc, HDLc, TG and glucose to baseline. Differences in laboratory variables between the baseline and at 12, 24, 36, 48 and 60 months PO were assessed using a one-way ANOVA test to compare the groups. Delta significance using one-way ANOVA was used to assess anthropometric variables PO. Statistical significance was defined as two-tailed p < 0.05. Statistical analysis was performed using SPSS (Statistical Package for the Social Sciences) software, version 21.0 for Windows.

## RESULTS

Of the 323 patients included in this study, 77 were included in G1 (24%), 101 in G2 (32%), and 141 in G3 (44%). Demographic data and the proportion of patients with abnormal laboratory values at baseline are shown in Table 1.

**Table 1:** Baseline characteristics of the patients who received RYGB over 5 years of follow-up

Number of patients (n = 323)	
Female	72% (233/323)
Male	29% (90/323)
Age (years)	38 ± 10
Height (m)	1,65 ± 0,86
Baseline Weight (kg)	123,91 ± 21,9
Baseline BMI (Kg/m^2^)	45,4 ± 6,1
TC > 200 mg/dL	40% (86/217)
LDLc > 130 mg/dL	31% (64/206)

BMI: body mass index, TC: total cholesterol; LDLc: Low-density lipoprotein cholesterol; HDLc: high density lipoprotein cholesterol; TG: triglycerides.

Table 2 showed no difference in the proportion of groups with abnormal values for the lipid subtractions and glucose concentration at baseline. Most patients from the 3 groups showed high TG before surgery.

**Table 2:** Laboratorial profile with abnormal levels at baseline

Laboratorial data	G1		G2		G3		p value
	n	Percentage	N	Percentage	n	Percentage	
TC > 200 mg/dL	19	36.5%	28	43.1%	39	39%	0.76
LDLc > 130 mg/dL	16	32%	20	32.8%	64	31.1%	0.90
HDLc < 40 mg/dL	16	32%	22	34.4%	30	30.9%	0.90
TG > 100 mg/dL	38	71.7%	53	82.8%	70	70%	0.17
Glucose > 100 mg/dL	15	28.8%	34	48.6%	43	41.7%	0.08

TC: total cholesterol; LDLc: low-density lipoprotein cholesterol; HDLc: high-density lipoprotein cholesterol; TG: triglycerides.

**Figure 1:**
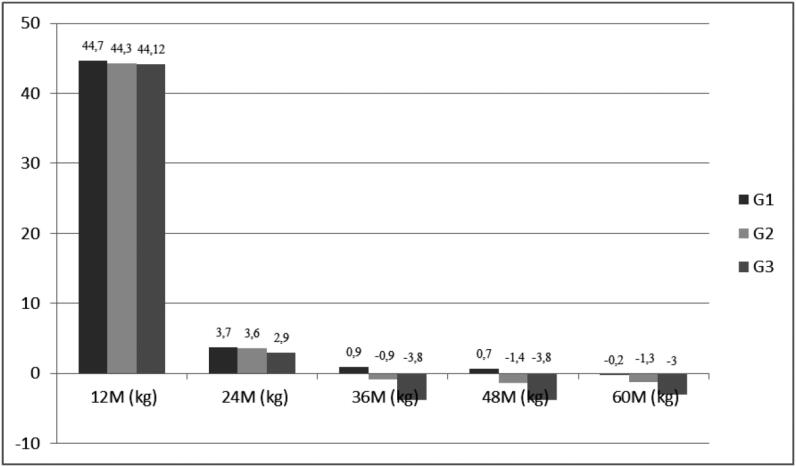
Weight loss (mean) in kilograms per year in patients who underwent RYGB. M: months; kg: kilograms; G1: group 1; G2: group 2; G3: group 3.

Figure 1 shows weight loss for G1, G2 and G3 during the postoperative period. Comparing these groups, a decrease in weight loss was observed in all groups followed by an increase in body weight for G2 and G3 at 36, 48, and 60 months (p < 0.01).

Laboratory results for G1, G2 and G3 are shown in Table 3. No differences between groups at baseline and during the post-operative period were found.

**Table 3:** Lipid profiles and glucose concentrations (means and standard deviation) of patients who underwent RYGB

Variable	Groups	Baseline		12 mPO		24 mPO		36 mPO		48 mPO		60 mPO	
		N	Mean/SD	n	Mean/SD	N	Mean/SD	N	Mean/SD	N	Mean/SD	n	Mean/SD
TC	G1	52	196±43	65	164 ± 33	56	170 ± 30	48	169 ± 43	20	164 ± 31	17	171 ± 32
	G2	65	200 ± 46	87	162 ± 28	77	170 ± 32	65	170 ± 34	44	170 ± 35	25	179 ± 32
	G3	100	200 ± 47	127	170 ± 34	107	170 ± 32	83	176 ± 36	71	180 ± 34	39	174 ± 41
	p value	--	0.85	--	0.18	--	0.99	--	0,48	--	0.12	--	0.77
LDL	G1	50	120 ± 35	64	93 ± 24	54	93 ± 22	48	84 ± 27	21	89 ± 28	17	89 ± 17
	G2	61	124 ± 43	86	90 ± 23	78	90 ± 27	65	90 ± 23	44	91 ± 27	38	89 ± 22
	G3	95	119 ± 36	125	95 ± 28	105	93 ± 24	81	95 ± 28	70	97 ± 30	88 ± 24	
	p value	--	0.70	--	0.45	--	0.66	--	0.07	--	0.38	--	0.98
HDL	G1	50	46 ± 10	62	56 ± 17	54	57 ± 14	48	65 ± 20	22	64 ± 15	19	67 ± 17
	G2	64	47 ± 15	86	56 ± 12	78	63 ± 17	64	64 ± 16	43	64 ± 16	25	68 ± 23
	G3	97	46 ± 11	123	55 ± 11	106	62 ± 15	80	67 ± 18	71	65 ± 20	41	61 ± 25
	p value	--	0.90	--	0.94	--	0.06	--	0.68	--	0.85	--	0.35
TG	G1	53	147 ± 70	62	87 ± 34	56	85 ± 37	46	83 ± 46	21	84 ± 35	17	89 ± 46
	G2	64	160 ± 93	86	78 ± 31	77	77 ± 29	63	78 ± 35	43	83 ± 41	25	84 ± 34
	G3	100	143 ± 63	125	86 ± 35	109	78 ± 32	82	78 ± 29	71	82 ± 34	37	86 ± 24
	p value	--	0.38	--	0.18	--	0.29	--	0.70	--	0.95	--	0.89
Glucose	G1	52	99 ± 20	63	83 ± 12	51	85 ± 8	44	83 ± 8	19	85 ± 8	14	89 ± 13
	G2	70	110 ± 36	87	85 ± 9	77	84 ± 8	58	87 ± 12	41	87 ± 12	21	89 ± 10
	G3	103	104 ± 29	127	83 ± 12	106	84 ± 6	78	89 ± 30	68	90 ± 32	35	86 ± 8
	p value	--	0.12	--	0.56	--	0.51	--	0.43	--	0.65	--	0.35

TC: total cholesterol; LDLc: low-density lipoprotein cholesterol; HDLc: high-density lipoprotein cholesterol; TG: triglycerides; 12 mPO: 12 months post-operative; 24 mPO: 24 months post-operative; 36 mPO: 36 months post-operative; 48 mPO: 48 months post-operative; 60 mPO: 60 months post-operative.

## DISCUSSION

This study examines the impact of weight regain on plasma glucose concentrations and lipid profiles in obese subjects during a 5-year follow-up period after marked RYGB-induced weight loss. The three groups of patients were comparable in terms of age, gender, anthropometric characteristics and laboratory results at baseline.

After the second or third year PO, long-term studies have shown a tendency for a weight increase in gastric bypass patients (15-20). In this study, weight loss did not differ significantly over the first 24 months after surgery.

Weight reduction can improve the lipid profile, alter carbohydrate metabolism, reduce morbidity and mortality, and generally enhance the quality of life of morbidly obese patients (21). Upper gastrointestinal tract diversion results in weight loss, but also may have weight loss-independent metabolic effects. Studies have reported contradictory findings regarding weight-loss dependent and independent effects of gastric bypass on glucose homeostasis and lipid profiles (1,8).

The RYGB technique may itself improve total cholesterol and LDLc levels (22). An observational study in obese patients confirmed that BMI is correlated with levels of TG and HDLc, but not with LDLc levels (23). In our study, glucose and lipid profiles were lower in all groups after surgery and this was not related to weight. Further studies analyzing laboratory parameters and weight dynamics after RYGB might contribute to clarifying whether the metabolic improvements seen after RYGB rely on weight loss or are an effect of the surgical technique itself.

The proportion of patients with abnormal total cholesterol concentrations prior to surgery was high in all groups but decreased during the long-term outcomes. Brolin and cols. (24) found that the greatest postoperative reductions in cholesterol and triglycerides generally occur in patients with the highest preoperative elevations, which is in accordance with our results for TG. Previous studies have shown a decline in total cholesterol and TG concentrations, and an elevation in HDLc concentrations, during the PO period. These changes were sustained over a long period of time in those patients who kept lost weight off as well as in those who experienced weight regain over time. Jamal and cols. (25) reported that the amelioration of the overall lipid profile was sustained over a 6-year PO follow-up.

Laguna and cols also showed that TG and LDL-C levels decreased 30% with respect to preoperative levels, while HDL-C increased 97% over initial values. Both TC:HDL-C and TG:HDL-C ratios normalized after RYGB and values were sustained over the weight regain period until the end of the study (26).

Bradley and cols. (6) support the idea that weight loss itself is primarily responsible for the clinical effects of RYGB on insulin sensitivity, β cell function, and oral glucose tolerance in non diabetic obese adults. On the other hand, Reed and cols. (27) showed improved fasting insulin and glucose levels a week after RYGB, despite persistent insulin resistance. Peterli and cols. (28) also reported early and augmented insulin responses as early as a week PO, potentially mediating improved early glycemic control. In our study, improved blood glucose levels were maintained throughout the postoperative period, regardless of weight regain. This may be associated with the enteroendocrine effects of RYGB, and suggests that glucose homeostasis after RYGB is weight-independent. Postoperative reduction in the levels of gastric inhibitory protein (GIP), and enhanced production of the antidiabetic hormone glucagon-like peptide (GLP-1), may underlie the changes in glucose seen after surgery (29). In addition, patients who participated in a prospective human study of gastric bypass had increased intestinal peptide YY (PYY) and GLP-1 responses, which were sustained even more than a year PO (30).

Numerous other factors have been implicated as potential contributors to the metabolic improvement observed after bariatric surgery, including other intestinal gut hormones (GLP-2, PYY), ghrelin (an anorexic hormone secreted by the gastric fundus), adipokines, the increased energy expenditure following surgery, changes in the gut microbiome, and bile acid metabolism (31).

Weight regain after gastric bypass has been attributed to multiple factors, including dilation of the gastric reservoir and gastrojejunostomy site, increased calorie intake of high glycemic index carbohydrates and fats, intolerance to red meat, hormonal alterations resulting from the adaptive process, binge eating, alcohol and drug consumption (32,33), difficulty promoting behavior changes, and lack of physical exercise. However, low adherence to follow-up with the multidisciplinary team PO is notoriously prevalent and problematic following bariatric surgery (34). Wardé-Kamar and cols. (35) suggest that such low adherence may occur due to underestimation of, or misinformation about, the surgery's consequences after long periods of time. Patients who regain weight may also feel embarrassed to seek help from the care service center where they had the operation. Reasons such as loss or change of health insurance, job loss, moving to another city or simply choosing not to continue the treatment after surgery may also reduce follow-up participation.

The limitations of our study include the retrospective nature of the analysis, incomplete follow-up, reduced sample size at the end of the study, and difficulty contacting patients who did not attend their scheduled follow-up appointments. These limitations are commonly reported in the literature for this kind of study. As laboratory results might change with further increases in body weight, a follow-up period of longer than 5 years would be necessary to establish whether they eventually return to preoperative levels (impairing the surgery's success and the patient's health). However, in spite of these limitations, long-term studies are crucial to determine long-term patient outcomes after bariatric surgery.

In conclusion, our results suggest that weight regain has no significant impact in the long-term evolution of the lipid profile and glycemia after RYGB. This study highlights the need for closer follow-up of RYGB patients, and for the implementation of actions aimed at improving adherence to the treatment protocols established by multidisciplinary teams. Additional long-term studies are required to establish whether a weight regain threshold might affect laboratory results, and to elucidate the exact mechanisms leading to these effects and their clinical consequences for the patient's health.
